# Sulphate Corrosion Mechanism of Ultra-High-Performance Concrete (UHPC) Prepared with Seawater and Sea Sand

**DOI:** 10.3390/polym14050971

**Published:** 2022-02-28

**Authors:** Xin Sun, Tianyu Li, Fangying Shi, Xiaoyan Liu, Yingxia Zong, Baorong Hou, Huiwen Tian

**Affiliations:** 1 Key Laboratory of Marine Environmental Corrosion and Bio-Fouling, Institute of Oceanology, Chinese Academy of Sciences, Qingdao 266071, China; sunxin@qdio.ac.cn; 2 University of Chinese Academy of Sciences, 19 A Yuquan Road, Beijing 100049, China; 3 College of Mechanics and Materials, Hohai University, Nanjing 210098, China; liuxiaoyan@hhu.edu.cn; 4 College of the Environment, Hohai University, Nanjing 210098, China; 2014010202@hhu.edu.cn; 5 College of Chemistry and Molecular Engineering, Qingdao University of Science and Technology, Qingdao 266071, China; zongyingxia@126.com

**Keywords:** sea sand, polymer cement mortar, UHPC, sulphate corrosion, material characterization, X-CT

## Abstract

The lack of river sand is becoming increasingly serious. In this study, we consider how to use sea sand to prepare innovative construction and building materials with excellent mechanical and durability properties. Sulphate corrosion causes expansion, cracking and spalling of concrete, resulting in the reduction or even loss of concrete strength and cementation force. In this paper, artificial seawater, sea sand, industrial waste, steel fiber and polycarboxylate superplasticizer were used to prepare ultra-high-performance polymer cement mortar (SSUHPC), and the sulphate corrosion mechanism was investigated. The strength and cementation force of mortar on the SSUHPC surface decreased and flaked off with the development of sulphate erosion, and the steel fiber rusted and fell off. A 3D model was established based on X-ray computed tomography (X-CT), and the results showed that SSUHPC maintained excellent internal structural characteristics despite severe sulphate erosion on the surface. Mercury intrusion porosimetry (MIP), scanning electron microscopy (SEM) and X-ray diffraction (XRD) techniques were adopted to investigate the sulphate corrosion mechanism of SSUHPC. We found a transition zone within 1–5 mm of the surface of SSUHPC. The Vickers hardness of mortar in this area was increased by 5~15%, and the porosity was reduced to 3.8489%. Obvious structural damage did not occur in this area, but a high content of gypsum appeared. UHPC prepared with seawater sea sand was found to have better sulphate resistance than that prepared with freshwater river sand, which supports the development and utilization of sea sand in concrete.

## 1. Introduction

Sulphate corrosion and chloride corrosion are the key factors of damage in concrete structures, which can result in reduced service performance and serious economic losses. The economic losses caused by sulphate corrosion are considerable worldwide, with annual repair costs in tens of billions to hundreds of billions of dollars (USD) [1]. UHPC has been widely used in engineering due to its ultra-high mechanical properties, durability and excellent service performance in various harsh service environments [2,3,4]. In this paper, we present a pioneering attempt to use seawater and sea sand to prepare UHPC and investigate is sulphate resistance.

Sulphate corrosion is defined as the destruction of cement concrete caused by a series of chemical reactions between sulphate ions from the external environment or concrete itself and cement hydration products. These changes may cause expansion and cracking, which reduce the strength and cementation force of concrete [5]. Depending on the type of cement hydration products and sulphate corrosion products, sulphate corrosion can be categorized as ettringite crystallization, gypsum crystallization, or MgSO_4_ dissolution–crystallization, etc. [6]. In the early stage of sulphate erosion, mainly aluminum-phase materials react with SO_4_^2−^ to form ettringite. SO_4_^2−^ easily diffuses and migrates to the surface of the aluminum phase, forming ettringite crystals on the surface of cement particles and causing structure expansion. Cracks appear on the surface of concrete, as erosion progresses further, spread deeper into the concrete. After ettringite forms on the surface, the eroded SO_4_^2−^ reacts with Ca^2+^ to form gypsum, consuming Ca(OH)_2_. The Ca^2+^ decomposed by Ca(OH)_2_ continuously forms gypsum with SO_4_^2−^. In order to maintain the balance with Ca^2+^ in the pore solution, the C-S-H gel begins to decalcify, resulting in decreased cementation force of the hydrated cement. Finally, the surface of the slurry appears to shed of its non-cohesive ability [7,8,9,10,11,12,13,14]. MgSO_4_ dissolution–crystallization is the most destructive of all sulphate erosion types on cement-based materials, with both Mg^2+^ and SO_4_^2^^−^ as erosion sources superimposed on each other to form compound erosion. Severe magnesium sulphate erosion will turns hydration products into paste without cementing properties. The main products formed under this type of erosion are brucite, ettringite, gypsum and hydrated magnesium silicate, etc., usually with a double-layer structure formed on the surface of the concrete. The outermost layer is brucite, with a thickness of 40–120 μm, and the inner layer is gypsum or other reaction products, with a thickness of 20–70 μm [15]. Large amounts of plate-like gypsum were generated by sulfate corrosion in freshwater river sand ordinary concrete [16]. The equations for the formation of ettringite and gypsum are as follows [5]:

$$C_{3}A+3C\bar{S}H_{2}+26H\to C_{6}A{\bar{S}}_{3}H_{32}$$

$$C_{4}AH_{13}+3C\bar{S}H_{2}+14H\to C_{6}A{\bar{S}}_{3}H_{32}+CH$$

$$C_{4}A\bar{S}H_{12}+2C\bar{S}H_{2}+16H\to C_{6}A{\bar{S}}_{3}H_{32}$$

$$Ca(OH{)}_{2}+{SO_{4}}^{2-}+2H_{2}O\to CaSO_{4}\cdot 2H_{2}O+{2OH}^{-}\overset{}{}$$

(1) $$C-S-H+2H_{2}O+{SO_{4}}^{2-}\to depleted\;C-S-H+CaSO_{4}\cdot 2H_{2}O\overset{}{}$$

$$M\bar{S}+CH+2H\to C\bar{S}H_{2}+MH$$

$$C-S-H+2M\bar{S}+5H\to 2MH+C\bar{S}H_{2}+S_{2}H$$

$$C_{4}AH_{13}+3M\bar{S}+2CH+20H\to C_{3}A\cdot 3C\bar{S}\cdot H_{32}+3MH$$

(2) $$2MH+S_{2}H\to 2M-S-H+H\overset{}{}$$

The following characteristics always appear in concrete structures that subjected to sulphate erosion [17,18,19]: (a) The concrete structure shows significant expansion and cracking; (b) some parts of the concrete structure are seriously corroded, resulting in obvious spalling of mortar and leakage of aggregate; (c) in low-temperature environments, mud-like and non-cementing materials may be formed, resulting in a significant reduction in the strength of concrete structure.

Much research has been conducted on the basic mechanical properties, impact properties, and microscopic change characteristics of sulphate-eroded concrete [20,21,22,23]. UHPC has been demonstrated to have excellent resistance to sulphate erosion. Sulphate erosion has little effect on the compressive strength and hydration-product microstructure of UHPC under different curing regimes [24]. Song [25] found that after 10 cycles of dry–wet sulphate erosion, the strength of UHPC was greatly improved, whereas ordinary concrete under the same condition was seriously damaged, which proves that UHPC exhibits resistance to sulphate corrosion. Sea-sand concrete plays an essential role in practical engineering and production. Much progress been made on chloride permeability and carbonization of sea-sand concrete [26,27,28]. In contrast, little research has been conducted on anti-sulphate erosion properties. Liu et al. [29] and Su et al. [30] conducted sulphate corrosion tests on desalinated sea-sand concrete and found that the loss of dynamic modulus of desalinated sea-sand concrete was slightly less than that of river-sand and sea-sand concrete, elucidating that desalinated sea-sand concrete has a slightly better sulphate corrosion resistance than river-sand and sea-sand concrete. Potential sulphate corrosion was observed in seawater sea-sand concrete, but the addition of active additives greatly improved its sulphate corrosion resistance [31]. Han et al. [16] formulated an environmentally friendly sulphate-resistant concrete using seawater sea-sand and high-ferrite Portland cement. Through the analysis of macro- and microstructures, it was discovered that seawater sand could increase sulphate resistance, mainly due to the refinement of pore structure and the consumption of erodibility components. Ting et al. [32] prepared concrete using silicomanganese slag, sea sand and seawater and reached a similar conclusion, improving the sulphate resistance of concrete.

X-CT technology, as a nondestructive testing technology, is widely used in construction and to observe internal microstructures of concrete [33,34,35,36,37]. Wang et al. [38] used X-CT to conduct 3D reconstruction of a UHPC cylinder sample and intuitively studied the spatial distribution of steel fibers and bubbles. X-CT technology has also been extensively used in sulphate corrosion research [39,40,41,42,43]. Internal pore space distribution and evolution of concrete in erosion environments are key issues in the study of material damage mechanisms and macroscopic mechanical properties. X-CT technology can intuitively exhibit microscopic pore changes in concrete under the combination of dry–wet cycles and sulphate erosion and establish connections among the macroscopic mechanical properties of concrete [40,44]. Tian et al. [45] conducted real-time scanning of the mesoscopic damage process of concrete under the coupling effect of sulphate corrosion and dry–wet cycles with X-CT technology, image processing and 3D reconstruction technology to elucidate the spatial distribution characteristics and evolution law of pore structures in concrete. The authors conducted an in-depth study of the mesoscopic damage characteristics and mechanisms of the materials. X-CT technology can also be used to study the 3D crack distribution of cement-based materials during sulphate corrosion. Yang et al. [41,46,47] used MIP, XRD and X-CT techniques to analyze the pore size distribution, corrosion products and three-dimensional crack distribution of samples, then studied the damage evolution of high-volume slag cement mortar exposed to sulphate erosion.

In this study, we used seawater sea sand and freshwater river sand to prepare ultra-high-performance concrete and systematically investigated the sulphate resistance of these two kinds of UHPC. The changes of external characteristics were studied by visual observation, and the changes of internal characteristics were studied by Vickers hardness and X-CT technology. We then compared the variation of these two UHPC materials under sulphate erosion. Mercury intrusion porosimetry (MIP), X-ray diffractometry (XRD) and scanning electron microscopy (SEM) were adopted to study changes in microscopic characteristics of UHPC materials in the process of sulphate corrosion, as well as the corrosion mechanism of UHPC materials under sulphate erosion.

## 2. Materials and Methods

### 2.1. Raw Materials

The materials used in this study include Ordinary Portland cement P·O 42.5 from Nanjing Conch Cement Co., Ltd. (Nanjing, China), silica fume from Gongyi Yuan Heng water purification material Factory, fly ash from Gongyi Yuan Heng water purification material factory, polycarboxylic acid series of superplasticizer from Shandong Yuncheng Brilliant New Building Materials Technology Co., Ltd., copper-plated straight steel fiber from Ganzhou Daye Metallic Fibres Co., Ltd., river sand and sea sand. The basic physical properties and chemical composition of the cement, silica fume and fly ash were determined by X-ray fluorescence (XRF) (Table 1, Table 2, Table 3 and Table 4). The water consumption for standard consistency and setting time of cement were tested according to Chinese standard GB/T1346-2019. The fineness of cement was tested according to Chinese standard GB/T 1345-2005. The density of cement was tested according to Chinese standard GB/T 208-2014. The compressive strength and flexural strength of cement were tested according to Chinese standard GB/T 17671-2021. The basic physical properties of fly ash were tested according to Chinese standard GB/T1596-2017. The basic physical properties of silica fume were tested according to Chinese standard GB/T 27690-2011. The steel fibers were 13 mm long, with a diameter of 0.2 mm, a length–diameter ratio of 65 and tensile strength greater than or equal to 2850 MPa. The solid content of superplasticizer was 40%, with a water-reduction rate of 35~40%. Sea sand was purchased from Zhangzhou, Fujian Province, with a fineness modulus of 2.3–2.6, a mud content of less than 1.0%, and a chloride ion concentration of 0.08%. The mud content of the river sand was 1.5%, with a fineness modulus of 2.2–2.5. An artificial seawater solution with a chloride concentration of 3.5% was prepared using analytically pure sodium chloride, composed of NaCl 24.53 g/L, MgCl_2_ 5.20 g/L, Na_2_SO_4_ 4.09 g/L, CaCl_2_ 1.16 g/L, KCl 0.695 g/L, NaHCO_3_ 0.201 g/L, KBr 0.101 g/L, H_3_BO_3_ 0.027 g/L, SrCl_2_ 0.025 g/L, and NaF 0.003 g/L, according to ASTM D1141-98. Either artificial seawater or fresh water was used as mixing water.

### 2.2. Mixtures, Specimen Preparation and Exposure Conditions

To investigate the effect of the use of seawater sea sand and freshwater river sand on the sulphate resistance of ultra-high-performance concrete (UHPC), two types of concrete were prepared, as shown in Table 5. Sand and steel fibers were added to cement, fly ash and silica fume and stirred for 3–5 min. Then, water and superplasticizer was added and stirred for 6–8 min. The concrete slurry was then loaded into the model and left for 24 h until follow-up treatment. SSUHPC refers to concrete made of simulated seawater and sea sand in 85 °C hot water and cured for 48 h. FRUHPC refers to concrete made of fresh water and river sand in 85 °C hot water and cured for 48 h. The specimens were Փ100 mm × 100 mm cylinders.

### 2.3. Test Methods

#### 2.3.1. Sulphate Corrosion Test

A demanding experimental condition was created in order to induce faster corrosion in the test environment. Sodium sulphate (33.8 g/L sulphate ion) and magnesium sulphate (33.8 g/L sulphate ion) solutions were prepared according to ASTM C1012. Then, the two solutions were mixed in equal volume to form sulphate compound solutions. Two kinds of concrete samples with a size of Փ100 mm × 100 mm were tested in the sulphate compound solutions at (25 ± 1) °C after curing, and the solution was changed every half month. The sulfate erosion test lasted six months, and the external change of the concrete material during sulphate corrosion was studied by visual observation.

#### 2.3.2. Compressive Strength and Flexural Strength

The compressive and flexural strength of the concrete was tested according to Chinese standard SL/T 352-2020. The compressive strength of samples with a size of 100 mm × 100 mm × 100 mm and the flexural strength of samples with a size of 100 mm × 100 mm × 400 mm were measured and repeated three times to obtain the average value.

#### 2.3.3. Vickers Hardness

Sulphate corrosion can cause severe strength deterioration, and the mechanical properties of concrete are critical to its performance. Therefore, we tested the mechanical properties of the samples. Irregular size changes appeared in the concrete after sulphate corrosion as a resulting cracking and falling off, making it impossible to accurately measure compressive strength. Thin subsamples of Φ50 mm × 10 mm were cut from different depths in the specimens for Vickers hardness measurements. Samples were cut and polished with abrasive paper to a standard thickness of 10 mm. In addition, to avoid the influence of humidity on the hardness of the concrete, the polished samples were placed in an oven at 45 °C for 24 h after the two end surfaces were polished. Vickers hardness was measured every 0.5 mm from the exposed surface to the interior of pastes using a micro-hardness tester (No. HDX-1000TC). The testing load was 0.980 7 N (0.1 kgf), and the applied loading time was 10 s. To obtain more representative results, eight data points of Vickers hardness were recorded at the same depth for each sample, uniformly distributed in a circle. Then, we removed the maximum and minimum values, and took the average of the remaining values as the representative value. The final Vickers hardness data were the average of three parallel samples.

#### 2.3.4. Scanning Electron Microscopy (SEM)

For the SEM test, we selected the same position of SSUHPC and FRUHPC before and after sulphate corrosion. Samples of the surface area were used to study morphology changes of the severely corroded area, and samples of the inner area were used to investigate the influence of sulphate corrosion on the interior of UHPC. All the samples selected for SEM testing were dried at 45 °C in an oven for 24 h and then coated with gold prior to testing. A Hitachi S-3400N scanning electron microscope was adopted. SEM images were photographed at 15 kV accelerating voltage. 

#### 2.3.5. X-ray Diffraction (XRD)

XRD was adopted to determined sulphate corrosion products. Samples were taken from the concrete surface and at depths of 1–3, 3–10, 10–20, 20–35 and 35–50 mm. XRD tests were conducted on a Bruker D8-Advance X-ray diffraction analyzer. Cu-Ka radiation was carried out with a wavelength of 1.54 Å under a current of 35 mA and a voltage of 40 kV. The scanning interval was 2θ = 5–65°, with a scanning speed of 2°/min and a step size of 0.02014°.

#### 2.3.6. Mercury Intrusion Porosimetry (MIP)

In order to understand the influence of sulphate corrosion on the pore structure of SSUHPC and FRUHPC, samples were characterized before and after corrosion using mercury intrusion porosimetry (MIP). The MIP test was performed in accordance with ISO 15901-1:2016. For this test, small cube samples of approximately 3 mm × 3 mm × 3 mm were cut from the new surface (1–3 mm) and at a depth of 3–5 (slight cross coverage of the sampling area). Small cube samples of approximately 5 mm × 5 mm × 5 mm were cut at depths of 5–10 mm, 10–15 mm, 15–20 mm, 20–35 mm and 35–50 mm. The samples were dried at 50 °C for 48 h in a vacuum oven. The MIP test was performed with an AutoPore IV 9510 mercury intrusion porosimeter (Micromeritics Instrument Corporation, Atlanta, Georgia, USA).

#### 2.3.7. X-CT Test and Data Analysis

A Siemens Somatom Sensation 40 CT scanner was adopted in this study to obtain mesostructural information about SSUHPC and FRUHPC, as well the composition and spatial distribution of ingredients. The SSUHPC and FRUHPC specimens were Փ100 mm × 100 mm cylinders. The X-ray CT system consisted of a radiation detector with a nominal resolution of less than 2 μm and a 240 kV/320 W microfocus X-ray source based on cone-beam scanning technology. The resolution of the microfocused X-ray source was 1 μm, and the minimum distance between the focus and the sample was 4.5 mm. A lamp voltage of 190 kV and a current value of 0.45 mA were adopted in this experiment. The cylindrical samples were secured to a table on a low-density polycylindrical base. In order to ensure beam uniformity, each specimen was automatically moved up and down during the 1 h scan.

**Figure 1:**
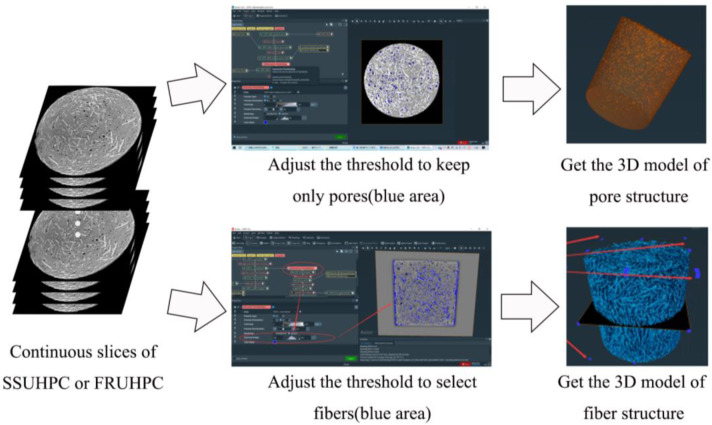
Schematic diagram of the reconstructed model of pore and fiber structure.

To analyze the pore and fiber structure of UHPC made of seawater sea sand and freshwater river sand, 3D models of the pore and fiber structure of each sample were developed based on continuous slices obtained by μX-CT. This process was performed with Avizo software. First, in order to obtain a 3D reconstruction model of the pore and fiber structure of each sample, thresholds were adjusted and selected to separate the pores or fibers from the solid structure. The pore and fiber structure statistics were obtained through a built-in function of the software for quantitative analysis. Figure 1 shows the schematic diagram of the reconstructed model of pore and fiber structure.

## 3. Results and Discussion

### 3.1. Compressive Strength and Flexural strength

**Figure 2:**
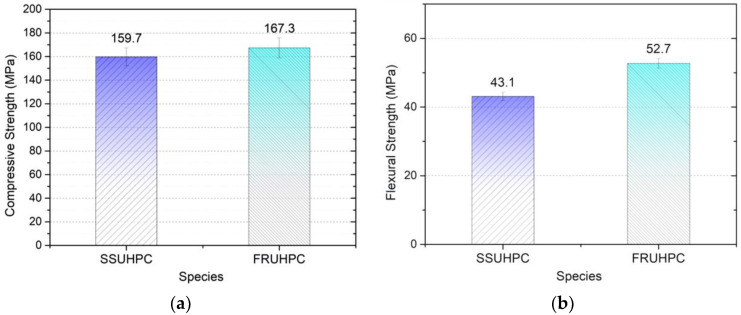
The mechanical properties of SSUHPC and FRUHPC. (**a**) Compressive strength; (**b**) flexural strength.

After curing, the mechanical properties of SSUHPC and FRUHPC concrete were tested. As shown in Figure 2a,b, the compressive strength of SSUHPC and FRUHPC was 159.7 and 167.3 MPa, and the flexural strength was 43.1 and 52.7 MPa, respectively. In this study, the compressive strength of two kinds of concrete was higher than 150 MPa [48], the index of ultra-high-performance concrete. Both kinds of tested concrete have excellent mechanical properties. The mechanical properties of UHPC prepared with seawater sea sand were slightly lower than those of UHPC prepared with freshwater river-sand.

### 3.2. Change of appearance

**Figure 3:**
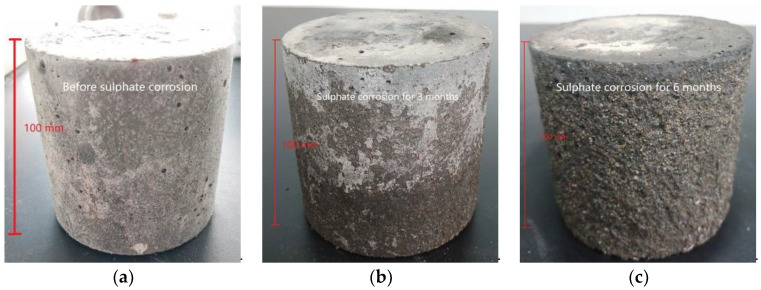

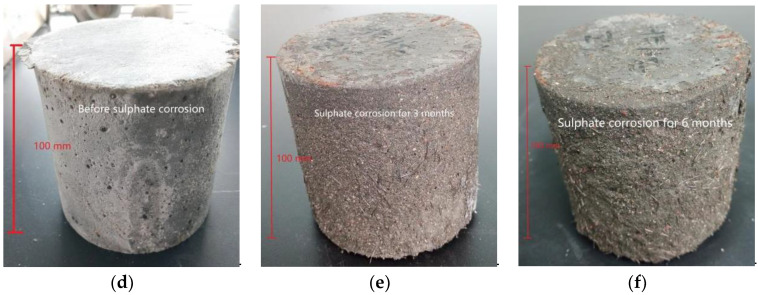
Appearance changes during sulphate corrosion. (**a**) SSUHPC, before sulphate corrosion; (**b**) SSUHPC, sulphate corrosion for three months; (**c**) SSUHPC, sulphate corrosion for six months; (**d**) FRUHPC, before sulphate corrosion; (**e**) FRUHPC, sulphate corrosion for three months; (**f**) FRUHPC, sulphate corrosion for six months.

Figure 3 shows the appearance changes of the two types of concrete during sulphate corrosion. After 3 months of sulphate corrosion, some areas of the SSUHPC surface became rough, although the overall surface did not change significantly compared the initial appearance (Figure 3a,b). After 6 months of sulphate corrosion, the concrete surface became coarse, and aggregate and pores on the surface area were exposed, as shown in Figure 3c. In contrast, the appearance of FRUHPC showed obvious changes after 3 months of sulphate corrosion. At this stage, the concrete surface became rough on the whole, and the aggregate along and pores on the surface area were exposed, as well as a large number of steel fibers (as shown in Figure 3e). After 6 months of sulphate corrosion, FRUHPC showed more serious sulphate corrosion characteristics. In addition to bare aggregates and steel fibers, obvious cracking and shedding occurred at this time (as shown in Figure 3f). On the whole, although these two concrete materials showed obvious sulphate corrosion damage, the damage of FRUHPC was more serious.

**Figure 4:**
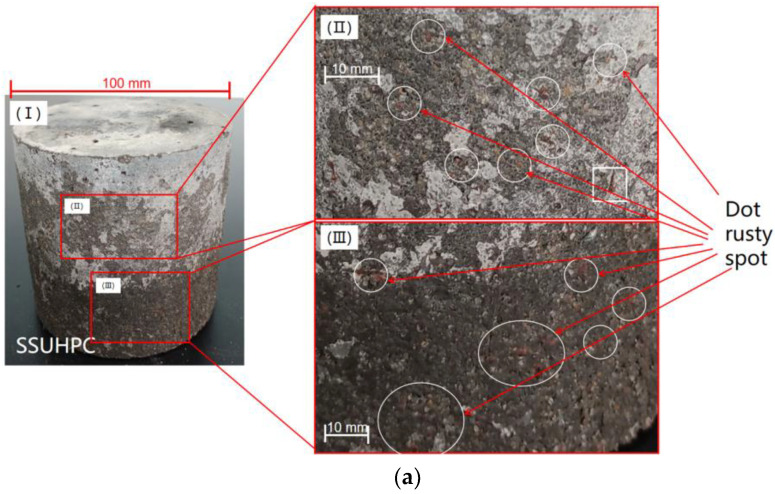

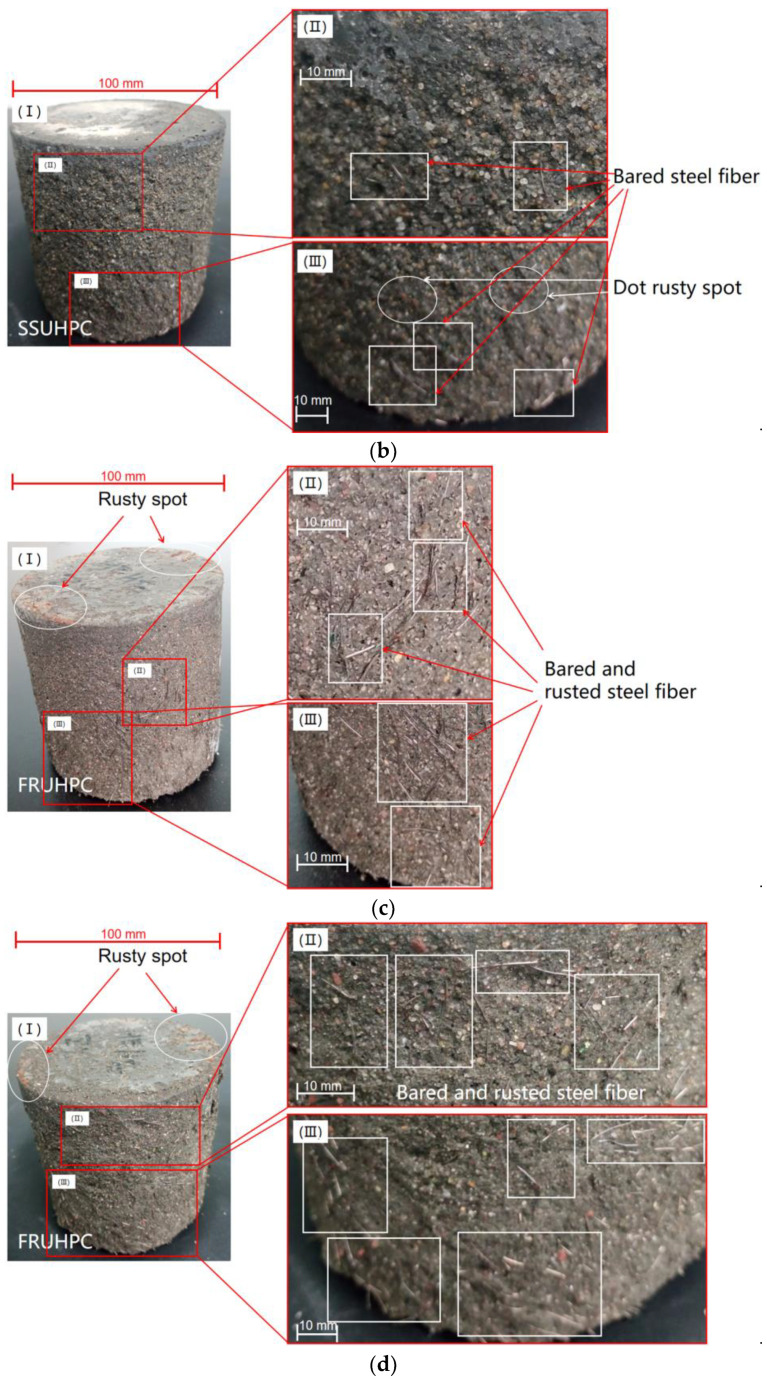
The corrosion characteristics of steel fiber after sulphate corrosion. (**a**) SSUHPC, sulphate corrosion for three months; (**b**) SSUHPC, sulphate corrosion for six months; (**c**) FRUHPC, sulphate corrosion for three months; (**d**) FRUHPC, sulphate corrosion for six months.

More detailed failure characteristics are shown in Figure 4, where we comparatively analyzed the degradation of steel fiber and deterioration of mortar. At the early stage of corrosion, sporadic brown rust spots appeared on the surface of SSUHPC. At this stage, in addition to the distributed rust spots, an area of seriously corroded steel fiber appeared on FRUHPC, as shown in Figure 4a,c. At the later stage of corrosion, a small number of corroded steel fibers appeared on the surface of SSUHPC. A large number of severely corroded, slightly corroded and uncorroded steel fiber were distributed on the surface of FRUHPC, as shown in Figure 4b,d. In the early stage of corrosion, some of the aggregate on the surface of SSUHPC was dissolved, and this change was gradually intensified with the progress of corrosion. In contrast, the dissolution phenomenon appeared to be serious in FRUHPC cement stone in the early stage of corrosion, and the concrete surface appeared to crack and peel with the progress of corrosion. Although the surface of SSUHPC was corroded and damaged, it maintained structural integrity. In comparison, the surface of FRUHPC was more seriously corroded, and a large number of surface areas that had not fallen off lost cohesiveness with the basal structure.

For SSUHPC, there was no significant morphological change in the early stage of corrosion, and the iron ions precipitated on the surface formed rust spots. With the progress of corrosion, the dissolution of the concrete surface was intensified, becoming rough with few steel fibers exposed. However, the sulphate corrosion damage of FRUHPC was more serious. The steel fibers in the surface area were exposed after the concrete surface was dissolved in the early stage of corrosion. With the progress of corrosion, the steel fibers exposed in the early stage peeled off, along with loose, cracking mortar, exposing new steel fibers. It was revealed from the appearance change of corrosion characteristics that UHPC prepared with seawater and sea sand has better sulphate resistance than that prepared with freshwater and river sand.

### 3.3. Internal Characteristics

Through visual observation, we can only obtain the change of exterior surface, but the characteristics of the concrete interior cannot be explored. In this section, X-CT technology was used to study the internal changes of SSUHPC and FRUHPC in the process of sulphate corrosion. The emphasis was on the analysis of mortar, pore structure and fiber structure.

#### 3.3.1. Variation of Cement Mortar

**Figure 5:**
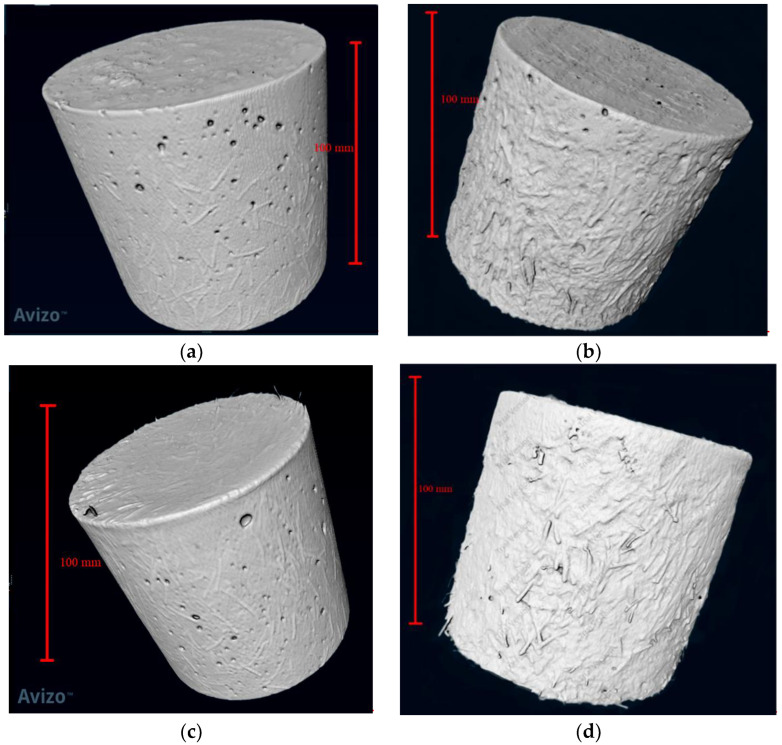
3D model of SSUHPC and FRUHPC obtained by Avizo software. (**a**) SSUHPC, before sulphate corrosion; (**b**) SSUHPC, sulphate corrosion for six months; (**c**) FRUHPC, before sulphate corrosion; (**d**) FRUHPC, sulphate corrosion for six months.

3D models of SSUHPC and FRUHPC were established by processing the slice data obtained from the X-CT test, as shown in Figure 5. The difference shown by the 3D model is that the concrete surface became rough after sulphate corrosion, which is consistent with the conclusion obtained through visual observation. In order to further investigate interior changes, we used a 3D model of the interior of the concrete for analysis.

**Figure 6:**
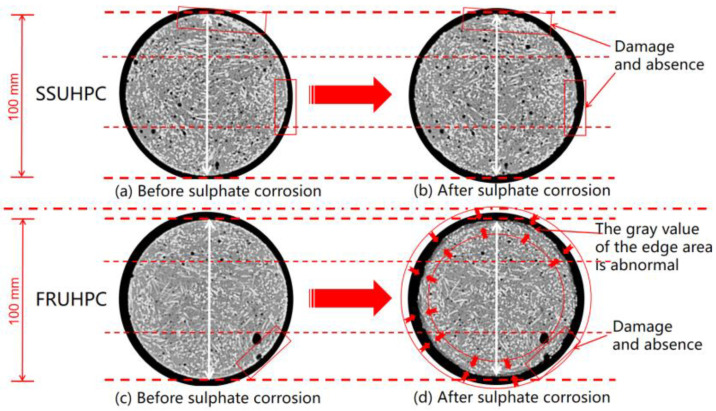
The internal damage of SSUHPC and FRUHPC during sulphate corrosion.

We select the same internal position in the SSUHPC and FRUHPC samples for a comparative analysis of internal changes before and after sulphate corrosion, as shown in Figure 6. After sulphate corrosion, the two kinds of concrete showed obvious damage and loss on the external surface but no change in the interior. SSUHPC and FRUHPC did not exhibit failure characteristics, such as expansion and cracking, under the action of sulphate erosion. At the same time, it can be seen that the gray value of FRUHPC’s peripheral area was higher than that of other internal areas after sulphate corrosion (see in Figure 6d), which indicates that the mortar properties of the concrete surface area had undergone obvious changes, although these changes did not appear in SSUHPC (see in Figure 6b).

**Figure 7:**
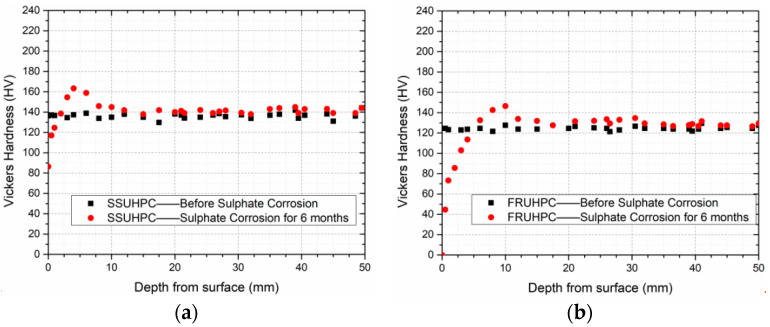
The variation in Vickers hardness versus depth from surface. (**a**) Vickers hardness of SSUHPC before and after sulphate corrosion; (**b**) Vickers hardness of FRUHPC before and after sulphate corrosion.

The Vickers hardness of SSUHPC and FRUHPC was tested before and after sulphate corrosion, and the test results were shown in Figure 7. After curing, the internal hardness of SSUHPC and FRUHPC were at the uniform level. With the progress of sulphate corrosion, the hardness of the concrete surface changed. After 6 months of sulphate corrosion, the hardness of mortar at a depth of 0–2 mm of SSUHPC was lower than the initial level, and that at a depth of 3–8 mm was significantly higher than the initial level. The hardness of mortar at a depth of 8 mm of SSUHPC was generally uniform, as shown in Figure 7a. Although the change rule of Vickers hardness of FRUHPC after corrosion was the same as that of SSUHPC, the hardness of mortar at a depth of 0–6 mm was obviously lower than the initial level, as shown in Figure 7b. The area of Vickers hardness significantly decreased in FRUHPC and was basically consistent with the area of gray value, which significantly changed (Figure 7b), indicating that significant sulphate corrosion damage had occurred in the concrete surface layer that has not fallen off.

#### 3.3.2. Pore and Fiber Structure Analysis Based on 3D Modeling

**Figure 8:**
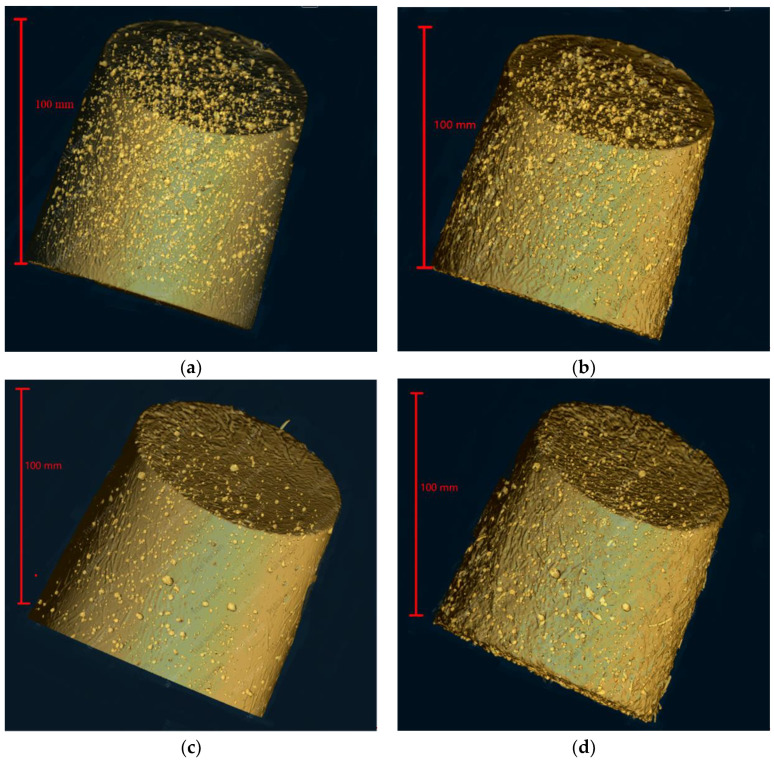
The change in pore structure between SSUHPC and FRUHPC during sulphate corrosion. (**a**) SSUHPC, before sulphate corrosion; (**b**) SSUHPC, sulphate corrosion for six months; (**c**) FRUHPC, before sulphate corrosion; (**d**) FRUHPC, sulphate corrosion for six months.

**Figure 9:**
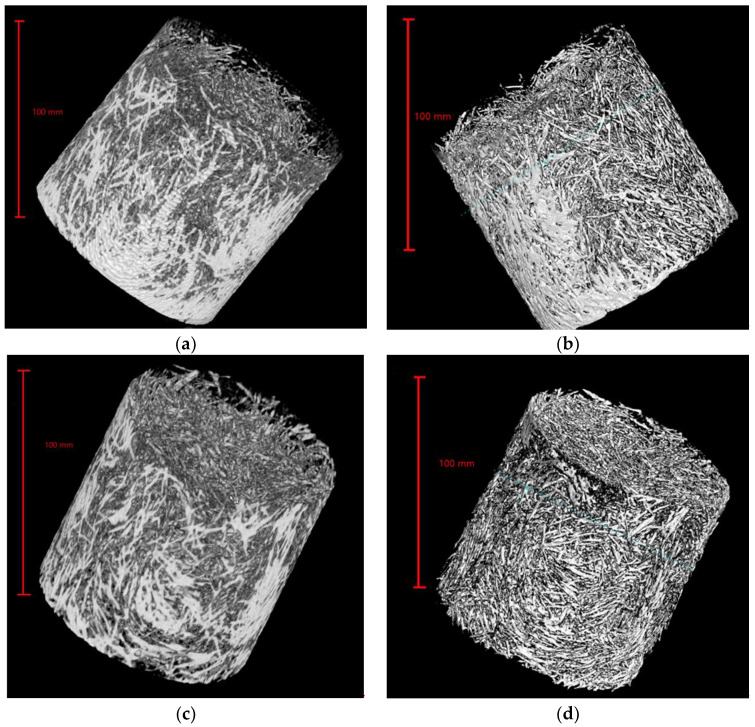
The change in fiber structure between SSUHPC and FRUHPC during sulphate corrosion. (**a**) SSUHPC, before sulphate corrosion; (**b**) SSUHPC, sulphate corrosion for six months; (**c**) FRUHPC, before sulphate corrosion; (**d**) FRUHPC, sulphate corrosion for six months.

The complete internal pore structure and fiber structure of SSUHPC and FRUHPC were obtained through modeling, as shown in Figure 8 and Figure 9. The pore structure established based on X-CT test results comprised mainly large pores and partial capillary pores. The number of internal pores in SSUHPC was significantly larger than that in FRUHPC, as shown in Figure 8a,c. However, the distribution and quantity of pores in the two groups of concrete were basically the same after sulphate corrosion, and the pore structure did not change, as shown in Figure 8a–d. Although steel fibers rusted and fell off in the surface area of the concrete during sulphate corrosion, the overall steel fiber structure inside the concrete did not change significantly, as shown in Figure 9. The model of pore structure and fiber structure showed that although the surface areas of the two kinds of concrete were severely corroded by sulphate corrosion, the internal area did not change, and the concrete material maintained its initial structural characteristics.

### 3.4. Microstructure Characterization

**Figure 10:**
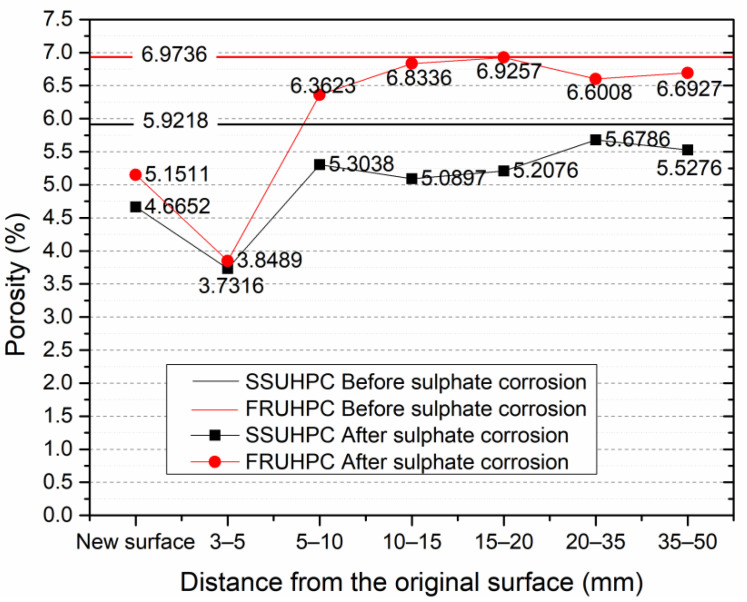
Change in porosity between SSUHPC and FRUHPC during sulphate corrosion.

In contrast with the pore structure model obtained on the basis of X-CT test results, the pore structure data obtained by MIP indicated capillary pores and gel pores in the mortar. A MIP test was conducted on the mortar of SSUHPC and FRUHPC before and after sulphate corrosion, and the test results are shown in Figure 10. Capillary pores and gel pores have the considerable influence on the mechanics and durability of concrete materials. After curing, the porosity of SSUHPC was 5.9218%, and that of FRUHPC was 6.9736%, which reveals, to a certain extent, why SSUHPC had improved sulphate resistance compared to FRUHPC.

After sulphate corrosion, the porosity of SSUHPC in the severely corroded surface area was reduced to 4.6652%, and that at a depth of 3–5 mm was reduced to 3.7316%. The porosity of the inner area was slightly lower than the relatively uniform initial porosity. The porosity change in FRUHPC after sulphate corrosion was basically the same as that of SSUHPC. The obvious difference was that the porosity at a depth of 3–5 mm dropped more dramatically, with a decreased from 6.9736% to 3.8489%. This indicates that more severe sulphate corrosion occurred in FRUHPC in this region, resulting in greater porosity changes. MIP test results showed that in addition to the obvious corrosion of steel fibers and the dissolution of cement stone on the concrete surface, the porosity of mortar at a depth of 1–5 mm also decreased obviously after sulphate corrosion. Although the porosity inside the concrete was also reduced, the reduction was less significant. This indicates that after sulphate corrosion, in addition to the outer surface corrosion, the remaining surface area of the structure also underwent significant corrosion change. We then studied the surface and interior changes of SSUHPC and FRUHPC by SEM and XRD.

**Figure 11:**
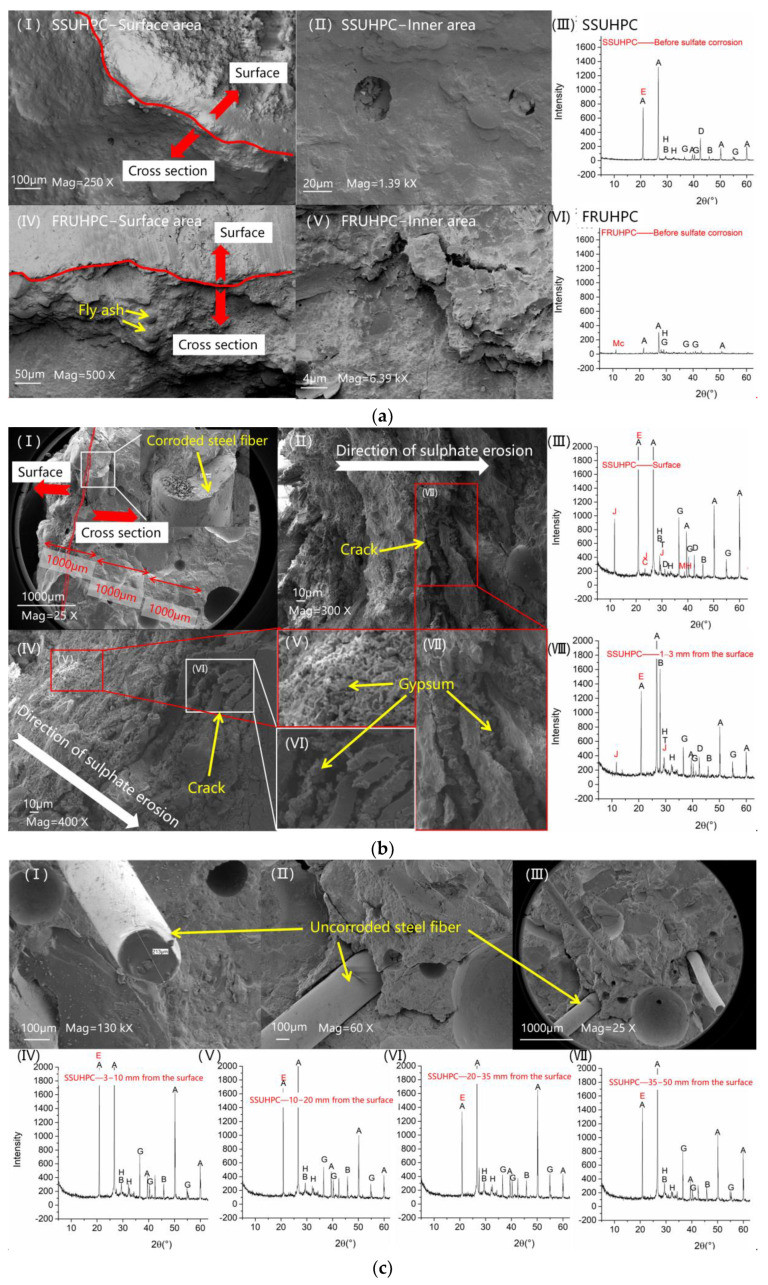

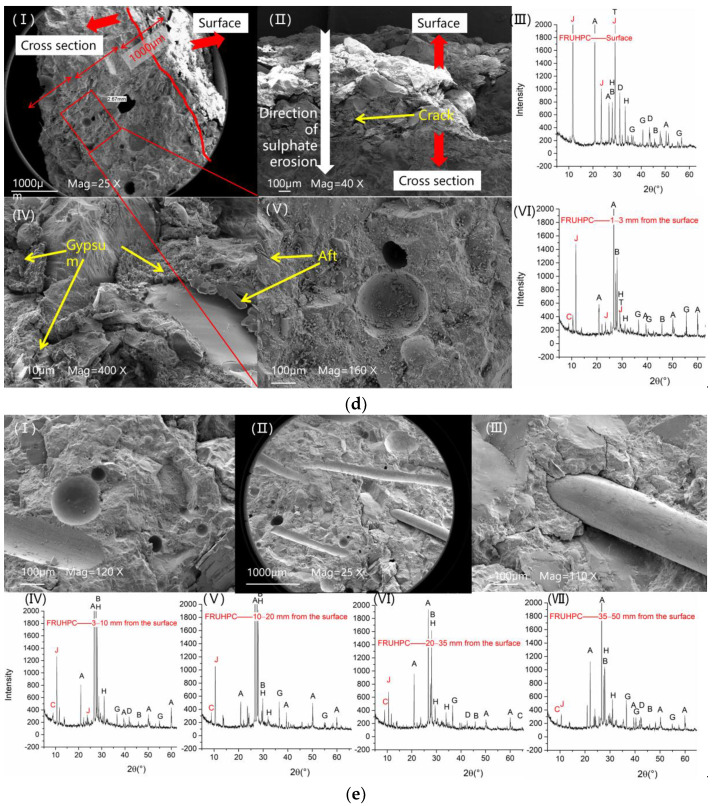
Changes in microstructural characteristics before and after sulphate corrosion. (**a**) Microstructural characteristics of SSUHPC and FRUHPC before sulphate corrosion; (**b**) surface area of SSUHPC after sulphate corrosion for six months; (**c**) inner area of SSUHPC after sulphate corrosion for six months; (**d**) surface area of FRUHPC after sulphate corrosion for six months; (**e**) inner area of FRUHPC after sulphate corrosion for six months. (A—SiO_2_; B—Ca(OH)_2_; C—AFt; D—CaCO_3_; E—Friedel’s Salt; G—C_3_S; H—CSH; J—Gypsum; MH—Mg(OH)_2_; T—talc_3_MgO·4SiO_2_H_2_O; Mc—Ca_4_AlCO_3_(OH)_12_·5H_2_O.

The changes in microstructural characteristics of the two kinds of concrete before and after sulphate corrosion are shown in Figure 11. The initial microscopic morphology and phase composition of SSUHPC and FRUHPC are shown in Figure 11a. The surface and internal area of the two samples show dense structural characteristics, and the filling effect of fly ash is also reflected in Figure 11(aⅣ). Because the design of UHPC adopted a low W/B ratio, there were unhydrated particles in the hydrated concrete, and C-S-H gel and C_3_S coexisted in the XRD maps of SSUHPC and FRUHPC. A diffraction peak of Friedel’s salt appeared in SSUHPC, which is the product of a combination of chloride ions in seawater sea sand and hydration products.

After corrosion, the surface area of SSUHPC was seriously damaged, and the phase composition of the microstructure became much more complex. At a depth of 1000 μm, the dense structure was seriously destroyed, and the cement stone was continuously cracked and broken off by corrosion products, as shown in Figure 11(bII,bIV,bVI,bVII). At this stage, steel fibers and aggregate located in the surface area of the concrete were exposed, and the steel fibers were seriously corroded, as shown in Figure 11(bI). XRD maps show strong diffraction peaks of gypsum and Friedel’s salt and a weak diffraction peak of AFt. At a depth of 1–3 mm, the microstructure remained dense and did not change significantly, and the XRD maps show that gypsum was still present in this area.

The structure and phase composition characteristics of the internal area of SSUHPC after corrosion are shown in Figure 11c. Compared with the initial state, the microstructure of the concrete did not change significantly after sulphate corrosion. The evenly distributed steel fibers showed no rust trace and were closely bonded with the dense mortar. Moreover, hydration products were evenly distributed in the structure near the surface area, although the same phenomenon did not occur in the structure near the center area, as shown in Figure 11(cI–cIII). XRD test results show that there was no diffraction peak of gypsum in the concrete, but the intensity of the diffraction peaks of Friedel’s salt and hydration products increased to a certain extent.

After corrosion, serious damage also occurred in the surface area of FRUHPC, as shown in Figure 11d. Compared with the surface area of SSUHPC, the changes in phase composition on the surface of FRUHPC were more significant, and the diffraction peak intensities of gypsum and C-S-H gel were rather remarkable, as shown in Figure 11(dⅢ). At a depth of 1–3 mm, the diffraction peak intensity of gypsum maintained a high level, and the diffraction peak intensity of Ca(OH)_2_ also increased abnormally, as shown in Figure 11(dⅥ). SEM test results also show that a large quantity of point-floccus-like gel, a mixture of sulphate corrosion products and rehydration products, was distributed in this region, as shown in Figure 11(dⅤ). The corrosion products were distributed sporadically, although the internal area of FRUHPC maintained a dense structure after corrosion, as shown in Figure 11e. XRD test results show that there were still diffraction peaks of gypsum and AFt in FRUHPC, which indicates that slight sulphate corrosion occurred inside the concrete.

### 3.5. Discussion

**Figure 12:**
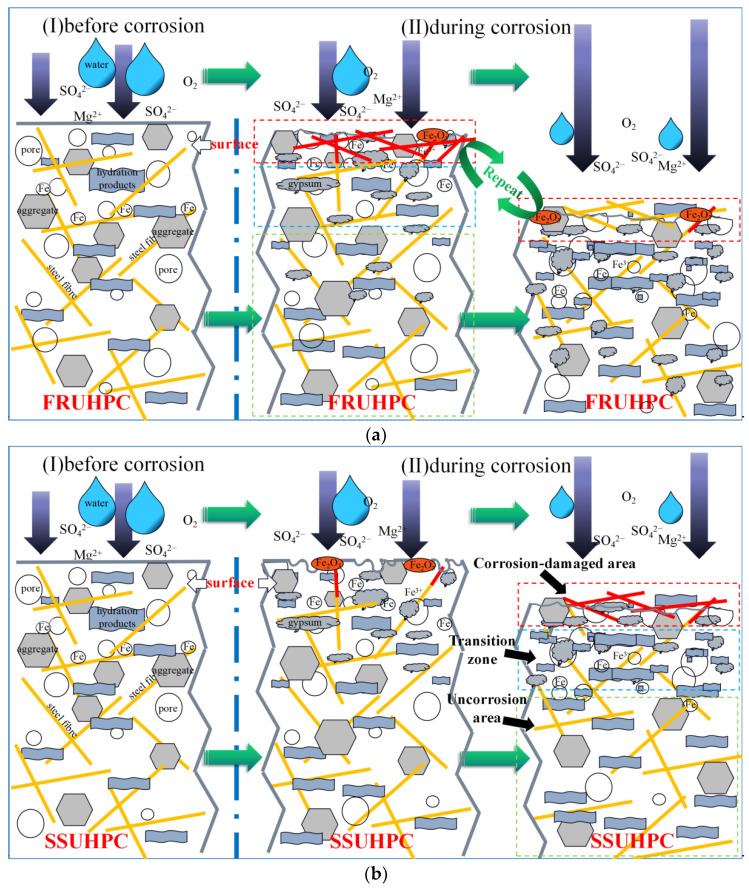
Schematic diagram of the sulphate corrosion mechanism of UHPC. (**a**) FRUHPC; (**b**) SSUHPC.

After 6 months of sulphate corrosion, both SSUHPC and FRUHPC showed obvious corrosion failure. For UHPC prepared with freshwater and river sand, corrosion damage appeared early under the action of sulphate. Hydrated cement stone on the surface of concrete reacts with Mg^2+^ and SO_4_^2−^ to generate sulphate corrosion products, such as AFt and gypsum. On the one hand, the emergence of a large number of corrosion products results in the consumption hydration products, such as Ca(OH)_2_ and C-S-H gels. On the other hand, the hydrated cement stone on the surface of concrete loses strength and cementation force and then falls off. The dissolution of the mortar exposes the aggregate and steel fibers located in the surface area, and the steel fibers rust when exposed to the environment. With the process of sulphate corrosion, sulphate corrosion products, such as AFt and gypsum, are generated. Mortar that loses strength falls off, along with steel fibers on the surface of the concrete, exposing the interior and forming a new exterior surface area. These two stages are repeated, causing the concrete surface to lose strength and cementation force and fall off layer by layer, as shown in Figure 12a. We found that the thickness of mortar for each peeling on the surface of UHPC was about 1–2 mm. Within a transition zone of about 5 mm adjacent to the surface of concrete, sulphate corrosion was in the primary stage, and the porosity decreased significantly under the simultaneous action of corrosion products and rehydration products. However, there was no obvious sulphate corrosion damage in the inner concrete. The macroscopic effect the sulphate corrosion of FRUHPC was that the outer surface was gradually destroyed at a depth of 1–2 mm, whereas the interior maintained excellent performance characteristics.

In contrast, UHPC prepared with seawater and sea sand showed better resistance to sulphate corrosion. Under prolonged sulphate corrosion, only slight cement dissolution occurred in some areas on the surface of SSUHPC. With the progress of corrosion, a small number of steel fibers were exposed and corroded. Subsequently, no significant peeling of mortar and steel fibers occurred until the end of the test, as shown in Figure 12b. Similarly to FRUHPC, serious sulphate corrosion damage occurred at a depth of about 1 mm in SSUHPC. A large amount of gypsum generated to deteriorated the dense concrete structure and exposed steel fibers, which were also seriously corroded. This area is called the corrosion-damaged area. There was also a transition zone adjacent to this zone, but in SSUHPC, the transition zone was thinner, with fewer corrosion and rehydration products than in FRUHPC. In contrast to the small amount of gypsum found in FRUHPC, no sulphate corrosion products were found in SSUHPC. Most importantly, SSUHPC did not suffer from the repeated damage and shedding of the external surface, as was the case for FRUHPC.

## 4. Conclusions

In this study, we compared the evolution of SSUHPC and FRUHPC under sulphate corrosion and summarized the sulphate corrosion mechanism. Our main conclusions are summarized as follows:

1. The evolution of UHPC under sulphate corrosion: (a) The cement stone in contact with sulphate was dissolved under the action of erosion, and the aggregate on the surface of the concrete was exposed and became rough. (b) As erosion developed, mortar on the surface of the concrete lost its cementation and gradually flaked off. Iron ions in steel fibers on the surface of UHPC were precipitated, and rust was formed on the surface. (c) With further sulphate erosion, the peeling degree of mortar on the surface was further aggravated, and the steel fibers on the surface were exposed and corroded. (d) With the continuous action of sulphate corrosion, the severely corroded steel fibers and weakened mortar with reduced cementation force on the surface of the concrete fell off. New mortar and unrusted steel fibers were exposed, and the process of (b)~(d) was repeated.
2. Compared with SSUHPC, FRUHPC was damaged more seriously by sulphate erosion. Vickers hardness results showed that the strength on the surface area (from 0 to 1 mm) of SSUHPC decreased, whereas the surface area of FRUHPC from 0 to 8 mm suffered a more serious loss of strength. Within a zone between the outer surface and the interior of SSUHPC and FRUHPC, the Vickers hardness of mortar was increased by 5~15%. Ultra-high-performance concrete made from seawater and sea sand was found to have better sulphate resistance than that made with fresh water and river sand.
3. X-CT test results showed that the structure of SSUHPC was relatively uniform, whereas the gray values of the outer surface and the inner surface in FRUHPC were obviously different. The concrete structure did not appear to exhibit volume expansion or cracking, and the pore structure and steel fiber structure did not show obvious changes. Although the surface of the concrete showed obvious spalling of mortar and steel fiber after sulphate corrosion, there was no obvious abnormality in the interior. Both SSUHPC and FRUHPC showed excellent sulphate resistance.
4. The surface area of UHPC was damaged, and spalling occurred with a 1~2 mm thickness due to sulphate corrosion. A large number of gypsum, Friedel’s salt and rehydration products appeared on the surface (within 1 mm) of SSUHPC and FRUHPC. Steel fibers were corroded and exposed on the surface of the concrete, presenting serious sulphate corrosion characteristics. There was no obvious structural damage at a depth of 1–5 mm, but a high content of sulphate corrosion products, such as gypsum, was present, and the porosity of SSUHPC was obviously reduced by 36.99%. However, there was no obvious abnormality inside the concrete, and the rehydration in this area was obvious.

## Figures and Tables

**Table 1:** Basic physical properties of cement.

Setting Time/min		Compressive Strength/MPa		Flexural Strength/MPa		Fineness/%	Density/(g/cm^3^)	Water Consumption for Standard Consistency/%
Initial Setting	Final Setting	3 d	28 d	3 d	28 d			
178	219	23.1	51.2	5.4	9.3	0.8	3.5	29.5

**Table 2:** Basic physical properties of fly ash.

Ratio of Water Demand/%	Fineness/%	Density/(g/cm^3^)	Compressive Strength Ratio/%	Specific Surface Area/(cm^2^/g)	Water Quantity/%	Packing Density/(g/cm^3^)	Normal Consistency/%
94	10	2.1	78	3400	106	0.78	48

**Table 3:** Basic physical properties of silica fume.

Density/(g/cm^3^)	Specific Surface Area/(cm^2^/g)	Average Particle Size/μm	Bulk Density/(g/mL)
1.6–1.7	(20–28) × 10,000	0.1–0.3	≥0.67

**Table 4:** Basic chemical composition of cement, fly ash and silica fume.

Species	Chemical Composition (wt.%)							
/	CaO	SiO_2_	Al_2_O_3_	MgO	Fe_2_O_3_	Na_2_O	SO_3_	Loss on Ignition
P·O 42.5 cement	61.536	15.404	4.430	0.724	4.906	0.043	2.755	2.243
Silica fume	0.568	97.35	0.337	0.414	0.003	0.101	0.192	2.810
Fly ash	1.5	58	30	2.8	4.3	3.2	0.8	3.310

**Table 5:** Composition of raw materials with different ratios (mass ratio).

Species	Cement	Fly Ash	Silica Fume	Steel Fiber	Polycarboxylic Acid Series of Superplasticizer	Sea Sand	River Sand	Artificial Seawater	Fresh Water
SSUHPC	0.6	0.25	0.15	0.19	0.03	1.4	\	0.14	\
FRUHPC	0.6	0.25	0.15	0.19	0.03	\	1.4	\	0.14

## Data Availability

The data underlying this article will be shared on reasonable request to the corresponding author.
